# The Babe of Bethlehem

**Published:** 1889-12-28

**Authors:** 


### Words of Consolation.

### THE BABE OF BETHLEHEM.

*(For Reading to the Sick.)*

Christmas has come, bringing with it messages of peace and goodwill. Our hearts glow within us when we think of the condescending mercy of the Father, who sent His Son into the world, and of the burning love of the Son, who for us men and for our salvation came down from heaven, and was made man. Here are reasons for endless rejoicing. And we will rejoice and be thankful, even though our bodies are cramped with pain and our heads bowed down with woe. For the child Christ came as at this time 1,800 years ago to find no welcome for Him, not even in an inn, therefore we will do our best to receive Him now into our hearts. We can never gaze too often on the beautiful picture set before us in the gospels of the trustful, loving Joseph guiding his tender young wife from Nazareth to Bethlehem, at the bidding of the Roman Emperor, to be taxed in their own royal city, because they were of David's line. Joyfully, as day drew to its close, at the end of their long journey, did they behold an inn, which they hoped would prove a shelter for her who was so soon to be a mother, and in which she might rest her weary limbs. But, alas! He came to His own, and His own received Him not. There was no room for them in the inn. He, the King of Kings and Lord of Lords, was thrust out into a stable, and there did not disdain to be laid among unclean beasts, even as He did not disdain to dwell for thirty years among the ungrateful Jews. But it was the clean manger in which He vouchsafed to rest his new-born limbs for the first hours of His life, in a place for food, in a place for refreshment; and if we keep ourselves clean and ready to receive Him, He will be our food, our refreshment, and our rest.

In a manger was laid the Babe of Bethlehem. What tender thoughts awake within us at those words, whether we think of Him as smiling on His mother and His earthly father, or as bearing the first woes which assailed His unaccustomed flesh! Who does not love a tender, innocent babe? How much dearer will it become when we know that that Babe is our Saviour and our King! Every Christian heart may be a cradle for the child-Christ. Let us, then, make preparations at once to receive Him into our own. First, we will sweep and cleanse our consciences by penitence and prayer, and then lay a firm faith for a foundation. His blessed head should be pillowed on sweet, loving deeds, and to these must be added gracious manners and courteous actions, a fitting covering for the cradle of the Lord. A Christian life should be lovely, and a true pleasure and delight to all who witness it. The crowning beauty is, however, the sweet grace of humility, which, like a curtain, casts a gentle shade around the heart where faith and love have made their dwelling-place. Shrinking from the world's rude gaze, it seeks but the Father's eye, and longs only to receive at the last the praise accorded to those who have done their best, "Well done, enter thou into the joy of the Lord."

And now our task is ended,
So haste we then to bring
The cradle we have furnished
For the glory of our King.

But yet a few words to those who have ere this prepared for their Lord's coming. Such may receive Him in their hearts still more closely by partaking of His most precious body and blood in the great sacrament of His love. By drawing near to Him in faith we become one with Him, we verily dwell in Him and He in us. He is the vine of which we are the branches. No branch can even live, much less bear ripe grapes, if it be cut off from the vine.

